# Cat Bite Cellulitis

**Published:** 2014-08-08

**Authors:** Nadia F. Nocera, Kunj K. Desai, Mark S. Granick

**Affiliations:** Division of Plastic Surgery, Department of Surgery, Rutgers New Jersey Medical School, Newark

**Keywords:** cat bite, hand cellulitis, lymphangitis, hand infection, *Pasteurella multocida*

**Figure:**
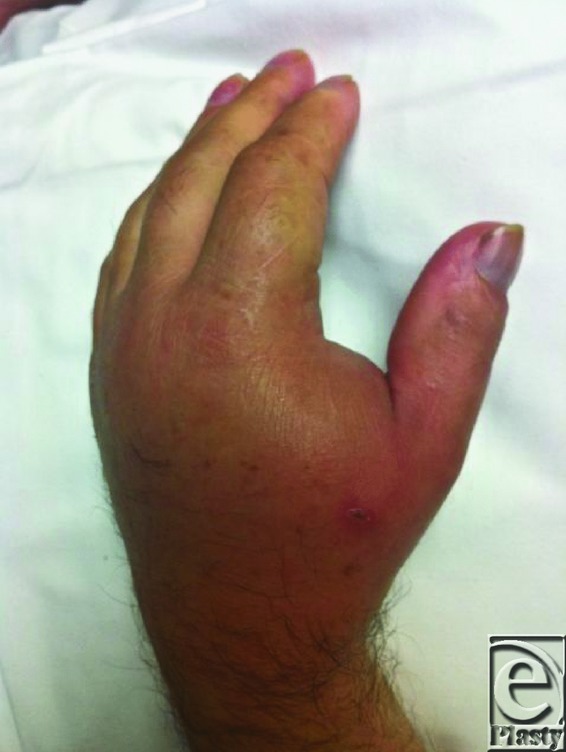


**Figure:**
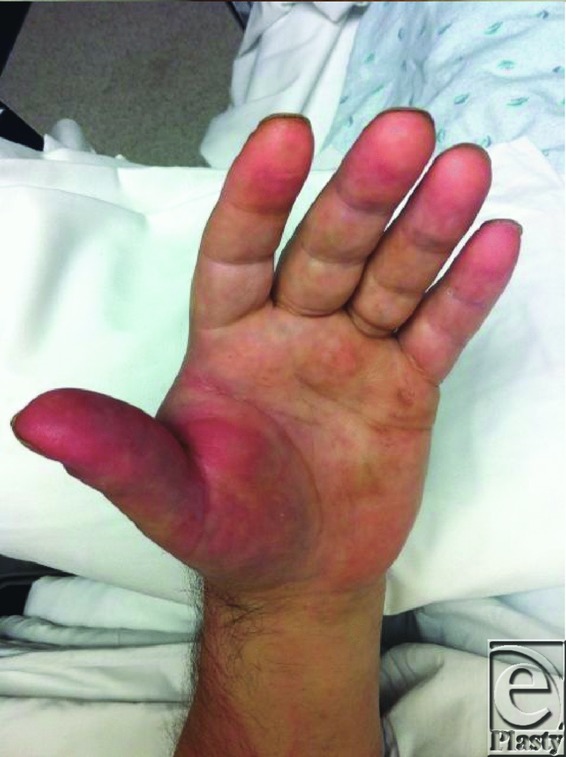


**Figure:**
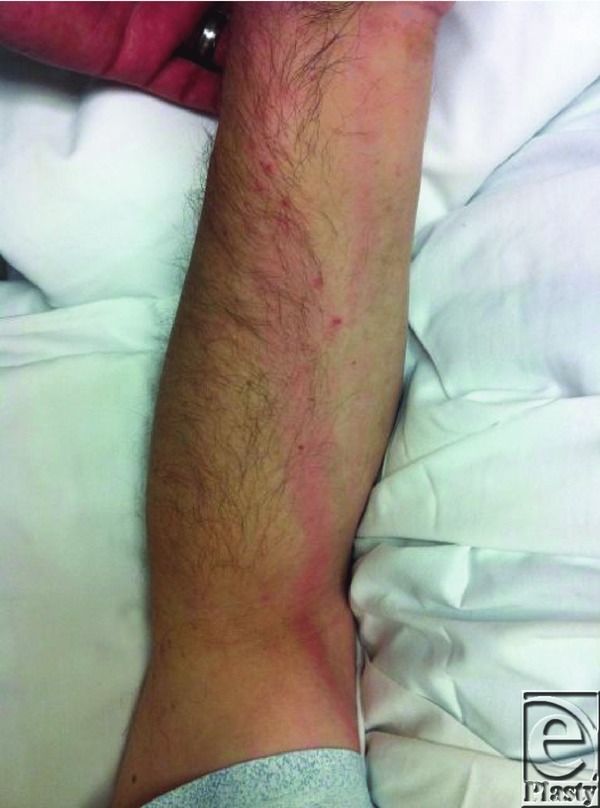


## DESCRIPTION

A 59-year-old man presents one week after sustaining a cat bite wound on the dorsum of the left hand. On examination, the left hand is swollen, erythematous and exquisitely tender on both dorsal and volar-radial aspects. Red streaking is noted extending proximally up the forearm and there is significant limitation of function. Active range of motion deficits are present at the wrist and thumb due to pain and swelling.

## QUESTIONS

1. **What is the potential risk associated with cat bites?**
2. **What are the common associated pathogens?**
3. **Describe the process of evaluating cat bite infections.**
4. **Discuss the treatment of cat bites according to severity of the injury.**

## DISCUSSION

Animal bites are a common entity among patient visits to the emergency department. In the United States, dog and cat bites comprise 1% of all emergency department visits. Cat bites represent approximately 3% to 15% of these cases.^1^^,^^2^ They are more likely to involve the upper extremity and face, with varying rates of infection ranging from 28% to 80%.^2^ Cats possess narrow, sharp teeth that can easily pierce the soft tissues like a hypodermic needle. This mechanism creates a small break in the skin that heals quickly, thus trapping the bacteria in the deeper tissues^4^ often resulting in invasive infection.^2^

Major groups of pathogens that have been isolated from cat bites including species of Pasteurella, Streptococcus, Staphylococcus, Neisseria, Corynebacterium, and Moraxella. Anaerobes included Fusobacterium, Bacteroides, Prevotella, and Porphyromonas species.^5^ Pasteurella has been found to be the most commonly isolated anaerobe in bites from cats, seen in 75% of cases, *Pasteurella multocida* being the most common species.^6^

Severe infections occur in about 20% of cases. The small enclosed compartments and fascial planes in the hand along with nerves, bones, and joints adjacent to the surface make the hand prone to developing deep space infections and osteomyelitis.^7^ Prompt evaluation and appropriate treatment of hand infections will allow for a favorable outcome without permanent disability. A detailed and thorough history including details of symptoms such as pain, loss of function, drainage, fever, and chills is necessary. The entire upper extremity should be examined for signs of cellulitis, deep space infections (abscesses), lymphadenopathy, and lymphangitis. A general physical examination to rule out systemic sepsis is also essential. Plain radiographs should be obtained for all bites. Magnetic resonance imaging/computed tomographic imaging are used to determine the presence of osteomyelitis or deep space infection when indicated.

While *P. multocida* sensitivity is well documented,^8^ it is not susceptible to many oral antibiotics routinely administered for skin and soft tissue infections such as cephalexin or clindamycin. Amoxicillin with clavulanate is the current recommendation for antibiotic treatment for cat bites.^9^ In penicillin-allergic patients or those with penicillin-resistant strains, other alternatives are necessary. Doxycycline with or without metronidazole or second- or third-generation cephalosporins (cefuroxime, cefpodoxime) can be utilized in such cases. Intravenous treatment is indicated when cellulitis and/or lymphangitis are evident, as in this case. This achieves detectable levels in the wound much sooner than by the oral or intramuscular route. Surgical intervention is indicated for drainage of any deep space infection, and primary closure of the bite wound is usually not necessary. Immunization against tetanus and rabies (where indicate) is advised.
